# The effect of 11th rib excision on perioperative outcomes in patients undergoing partial nephrectomy for upper pole renal tumors

**DOI:** 10.1007/s11255-024-04087-5

**Published:** 2024-05-24

**Authors:** Burhan Baylan, Abdullah Gurel

**Affiliations:** Department of Urology, Afyonkarahisar Health Sciences University, Zafer Sağlık Külliyesi Dörtyol Mah. 2078 Sokak, No: 3, A Blok, Pk, 03030 Afyonkarahisar, Turkey

**Keywords:** Kidney tumor, Partial nephrectomy, Rib resection, Renal ischemia

## Abstract

**Introduction:**

We aimed to evaluate the effect of eleven11th rib resection.on the perioperative period TRIFECTA criteria in patients who underwent retroperitoneal partial nephrectomy (PN) with the diagnosis of upper pole kidney tumors.

**Materials and methods:**

We conducted a retrospective analysis of the data of the patients who underwent Open PN for upper pole renal masses between 2018 and 2023. The patients were divided into two groups: PN with rib resection and PN without rib resection. The demographic characteristics, tumor sizes, PADUA scores, warm–cold renal ischemia times, mass excision and tumor bed suturing times, histopathological tumor type and surgical margin positivity of the patients were examined. Both groups were evaluated comparatively based on this data.

**Results:**

The renal nephrometry scores of the two groups were similar. The total renal ischemia time was significantly shorter in the patients who underwent a rib resection than in those who did not (*p* < 0.001). Both the tumor excision and tumor bed suturing times were significantly shorter in the group that underwent a rib resection than in the group that did not (*p* < 0.001). The Clavien–Dindo complication grades were statistically similar between the two groups.

**Conclusion:**

Complex in nature and high–risk renal masses located in the upper pole of the kidney, partial nephrectomy performed with an 11th rib resection can be considered a reliable surgical option with a shorter ischemia time, supporting the preservation of long-term renal function.

## Introduction

The increasing use of computed tomography (CT) and magnetic resonance imaging (MRI) in recent years has led to an increase in the detection rate of small-sized renal masses, and in parallel to this, changes have been witnessed in the preferred surgical treatment approaches [1].

Nephron-sparing surgery, or partial nephrectomy (PN), is recommended by many international guidelines for the surgical treatment of small renal masses [2, 3]. Clinical stage T1a lesions are best managed by PN, although there have been suggestions that PN may be preferable also for larger renal masses. The European Association of Urology recommends partial nephrectomy for T1b lesions, while the American Urological Association recommends radical nephrectomy [2, 3]. The preferred surgical techniques for partial nephrectomy are transitioning from open procedures to minimally invasive laparoscopic and robotic surgeries, driven by technological advances. While recent studies indicate that laparoscopic and robotic-assisted approaches yield comparable oncologic outcomes to open surgery [2], open partial nephrectomy remains an appropriate surgical option for high-risk tumors.

The TRIFECTA criteria have been defined for patients undergoing partial nephrectomy, namely negative surgical margins, minimal warm ischemia time (WIT) (less than 25 min) and the absence of postoperative complications, regardless of the surgical technique employed [4–6]. Although it has been advocated that the ischemia time should be less than 25 min in the TRIFECTA criteria, the general consensus emerging from many studies is that “time means tissue” and “every minute counts” in efforts to reduce the risk of long-term renal failure [7, 8].

In partial nephrectomies performed using a retroperitoneal approach for tumors located in the upper pole of the kidney, difficulties may arise both during tumor excision and in subsequent tumor bed suturing, and in parallel to this, the ischemia time may be prolonged, especially in cases requiring ischemia. With this hypothesis in mind, the present study assesses the perioperative period based on the TRIFECTA criteria in a study of patients undergoing retroperitoneal partial nephrectomy for upper pole kidney tumors using a flank incision, along with rib resection.

## Materials and method

After the study was granted ethical approval by the Afyonkarahisar Health Sciences University Clinical Research Ethics Committee (2024/79), a retrospective review was carried out of patients who underwent open retroperitoneal partial nephrectomy for upper pole renal masses in the Department of Urology of Afyonkarahisar Health Sciences University between 2018 and 2023. The patients undergoing open partial nephrectomy were divided into two groups: those who underwent a concurrent rib resection (PN with rib resection); and those who did not undergo a concurrent rib resection (PN without rib resection). The demographic characteristics, tumor sizes, PADUA scores, operation times, warm–cold renal ischemia times, mass excision and tumor bed suturing times, estimated blood loss, and transfusion requirements of the two groups were recorded, and the pathologic tumor size, histopathological tumor type and surgical margin positivity of the patients were examined. Both groups were evaluated comparatively based on the collected data.

**Fig. 1:**
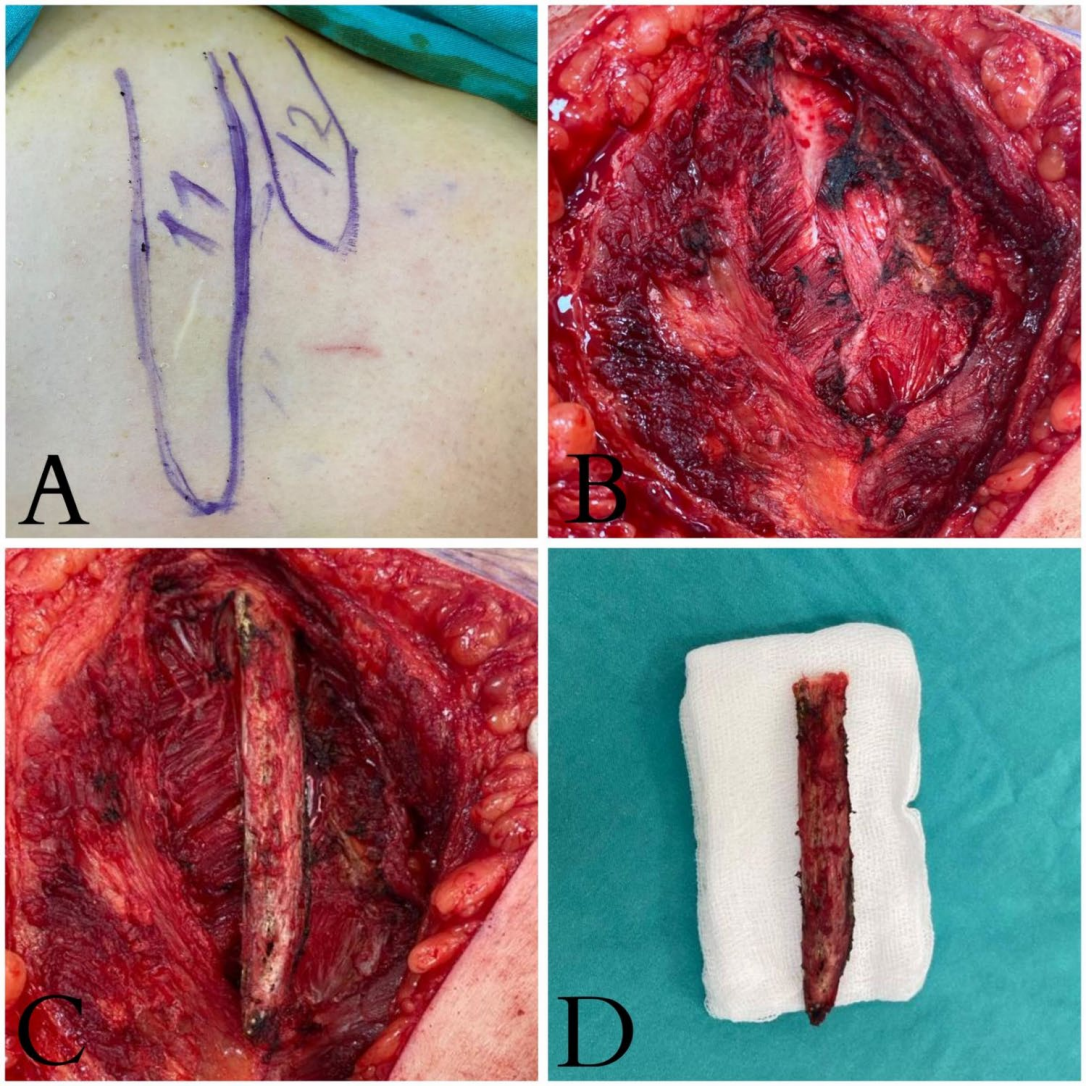
Surgical images of 11th rib excision

Surgical technique for 11th rib resection: under general anesthesia, the patient is placed in the flank position and the 11th rib is localized and marked (Fig. 1A). The skin and subcutaneous muscle layers are incised over the 11th rib to expose the rib (Fig. 1B). The periosteum layer over the rib is dissected from the rib, leaving the periosteal bed intact, and the 11th rib is mobilized proximally and elevated (Fig. 1C). In the final step, the rib is excised and removed using a rib cutter (Fig. 1D). Irregular protrusions along the sharp edge of the excised area are smoothed using a rasp. Deep inspiration is then induced in all patients using a mechanical ventilator to check for any pleural defects after resection.

### Statistical analysis

The data analysis was conducted using the IBM SPSS Statistics (Version 25.0. Armonk, NY: IBM Corp.). The normality of the distribution of continuous variables was assessed with a Shapiro–Wilk test, while Levene’s test was employed to examine the assumption of homogeneity of the variances. Descriptive statistics included mean ± standard deviation (SD) for continuous variables, while categorical variables were expressed as numbers and percentages. After conducting goodness-of-fit tests, the significance of the differences between the groups in the continuous variables that met parametric test assumptions was assessed with a Student’s *t* test, while the differences in continuous variables that did not meet the parametric test assumptions were analyzed with a Mann–Whitney *U* test. For the analysis of categorical data, Fisher’s exact test was utilized if the expected frequency in at least ¼ of the cells in 2 × 2 contingency tables was less than 5. In cases where the expected frequency was in the 5–25 range, a Continuity-corrected *χ*^2^ test was employed. In the RxC contingency tables (in which at least one of the categorical variables in the rows or columns has more than two outcomes), if the expected frequency in at least ¼ of the cells was less than 5, the categorical data were assessed using a Fisher-Freeman-Halton test.

## Results

A total of 48 patients underwent partial nephrectomy for upper pole renal masses, of whom 26 underwent a retroperitoneal partial nephrectomy via a flank incision below the 12th rib without rib resection, while 22 patients underwent a retroperitoneal partial nephrectomy after the 11th rib was resectioned. A rib resection and partial nephrectomy were performed in all cases by a single uro-oncology surgeon. Both groups comprised patients who underwent surgery for upper pole renal tumors. The median age was 61.3 years in the patients undergoing PN without rib resection, and 59.4 years in those undergoing PN with rib resection. The median tumor size was 4.9 cm (range: 3.9–6.8) in patients undergoing PN without rib resection, and 4.6 cm (range: 3.5–7) in those undergoing PN with rib resection. The renal nephrometry scores of the two groups were similar (see Table 1).

**Table 1:** Demographic characteristics

	PN without rib resection (*n* = 26)	PN with rib resection (*n* = 22)	*P* value
Age (median)	61.3	59.4	0.024**†**
Sex			
Male	10	8	0.116‡
Female	16	14	0.128‡
Tumor side			
Right	14	12	0.678¥
Left	12	10	
Tumor size *	4.9 (3.9–6.8)	4.6 (3.5–7)	0.318
Renal nephrometry score			
4–6	4	4	0.380‡
7–9	12	10	0.283‡
10–12	10	8	0.151‡

Descriptive statistics were expressed as *medians (minimum–maximum). †Student’s t test, ‡χ^2^ test with continuity correction, ¥Fisher-Freeman-Halton test

Among the patients who did not undergo a rib resection, four did not undergo ischemia, while 14 underwent cold ischemia and eight underwent warm ischemia. Among the patients who underwent a rib resection, four patients did not undergo ischemia, while 12 underwent cold ischemia and six underwent warm ischemia. The two patient groups were comparable in terms of the preferred ischemia methods. The total renal ischemia time was significantly shorter in the patients who underwent a rib resection than in those who did not. (*p* < 0.001) Both the tumor excision and tumor bed suturing times were significantly shorter in the group that underwent a rib resection than in the group that did not. (*p* < 0.001).

The median operation time was 96 min (range: 78–126 min) in the non-rib resection group and 104 min (range: 82–132 min) in the rib resection group. The median estimated blood loss was 482 ± 172 ml in the non-rib resection group and 420 ± 156 ml in the rib resection group. Intraoperative blood transfusions were administered to one patient in both groups. The median tumor size was 3.69 cm (range: 1.8–7.1 cm), and the median preoperative and postoperative hemoglobin levels were 144 and 112 g/L, respectively (*p* = 0.19), with a median decrease in hemoglobin levels of 31 g/L. A comparison of the median preoperative and postoperative creatinine levels revealed a mean increase of 20 µmol/L in the group non-rib resection group and 18 µmol/L in the rib resection group (*p* = 0.05). The Clavien–Dindo complication grades were statistically similar between the two groups. There was no need for reoperation or complementary nephrectomy in either group. In the non-rib resection group, two Grade 2 (Clavien–Dindo) complications and one major complication (Clavien IIIa) were observed, while in the rib resection group, three Grade 2 complications and one major complication (Clavien IIIa) were observed (Table 2).

**Table 2:** Perioperative and postoperative outcomes

	PN without rib resection (*n* = 26)	PN with rib resection (*n* = 22)	*P* value
Renal ischemia type			
Non-ischemic	4	4	0.325‡
Cold ischemia	14	12	
Warm ischemia	8	6	
Renal ischemia time (min)*	25 (15–34)	14 (11–20)	** < 0.001**
Mass excision time (min)**	3.6 ± 0.7	1.1 ± 0.3	** < 0.001**
Tumor bed suturing time (min)**	20.1 ± 8.3	12.8 ± 4.5	** < 0.001**
Operation time (min)*	96 (78–126)	104 (82–132)	0.795
Estimated blood loss (ml)**	482 ± 172	420 ± 156	0.636
Postoperative blood transfusion	3 (11.5%)	2 (9%)	
Postoperative increase in creatinine levels (µmol/L)	22	18	0.05
Clavien–Dindo classification of complications			
Grade 1	4	5	0.116‡
Grade 2	2	3	
Grade 3a	1	1	

Descriptive statistics were expressed as *medians (minimum–maximum) or **means ± standard deviations. †Student’s t test, ‡χ^2^ test with continuity correction

Clinically significant statistical data are in bold

The histologic values of the tumors in both groups are presented in Table 3. In the non-rib resection group, three patients were identified as pathologic stage 3a, while in the rib resection group, one patient was identified as pathologic stage 3a. Surgical margin positivity was observed in two patients in the non-rib resection group and in one patient in the rib resection group.

**Table 3:** Pathologic tumor data

	PN without rib resection (*n* = 26)	PN with rib resection (*n* = 22)
Pathologic tumor type		
Clear cell	12	10
Chromophobe	6	5
Papillary type 1	2	4
Papillary type 2	1	2
Multicystic renal cell carcinoma	1	0
Oncocytoma	2	1
Angiomyolipoma	2	0
Surgical margin positivity	2	1
Pathologic stage		
T1a	12	11
T1b	12	10
T3a	2	1

## Discussion

PN has become the standard surgical approach for the removal of small-sized renal masses and cT1b renal lesions. The increased use of imaging modalities has led to an increase in diagnoses of renal masses, for which partial nephrectomy continues to be the preferred technique among surgical oncologists [2]. There remains a lack of consensus on the most appropriate surgical option for patients requiring PN among the robotic-assisted, laparoscopic or open options. Although laparoscopic and robotic-assisted procedures are frequently employed, the open surgical approach is technically easier and safer when encountering complex tumors. The traditional or large flank incision is often made superior to the 12th rib, as it provides the opportunity to work within the retroperitoneum while avoiding intraperitoneal structures. This approach typically involves the resection of the distal one-third of the 11th or 12th rib, but can be completed in a coordinated manner without resection [9, 10].

The duration of ischemia during partial nephrectomy is recognized as crucial for the long-term preservation of renal function [11]. Thompson et al. reported on the impact of warm ischemia on both the short- and long-term preservation of renal function. Progressive WIT has an impact on the incidence of acute kidney injury or short-term renal function, while another prognostic factor for impairment in renal function is tumor size. The authors suggest that the extensive suturing of healthy parenchymal tissue could have a detrimental effect, and resected kidney tissue expands as the tumor size increases in such patients [12].

Previous studies have suggested that the WIT should be kept below 25 min in cases of partial nephrectomy [13]. Observational studies have reported a WIT of less than 20 min to be a strong modifiable, protective factor, stating that each additional minute of WI above 20 min is associated with a significant decrease in the estimated glomerular filtration rate (eGFR) [11, 14].

The 20 min threshold is currently accepted as optimum [15]. Our study differs from previous research in terms of its scope, in that there has to date been no study of patient groups undergoing partial nephrectomy with rib resection. The median ischemia time in the present study was found to be 25 min (range: 15–34 min) in the non-rib resection group, compared to 14 min (range: 11–20 min) in the rib resection group – meaning a significantly shorter ischemia time in the group of patients who underwent PN with rib resection.

Previous studies in literature have reported transfusion rates for PN in the range of 4–20% [16], and similar rates to those reported in literature were identified in the present study, with two patients (9%) requiring transfusion in the rib resection group and three patients (11.5%) in the non-rib resection group, with no statistically significant difference between the two groups in this regard.

Diblasio et al. and Wang et al. reported better oncologic outcomes, fewer wound complications and better cosmetic results in mini OPN cases in which an incision measuring 8–10 cm was made above the 11th rib [17]. In the present study, no significant difference was observed between the two groups (those who underwent a rib resection and those who did not) in terms of wound complications. Furthermore, in the present study, rib resection procedures were not associated with any complications requiring additional surgical interventions in either the intraoperative or postoperative periods.

The limitations of our study include its retrospective nature and small sample size. These constraints should be considered when interpreting the findings, and future prospective studies.

## Conclusion

Open partial nephrectomy continues to be an appropriate surgical option in cases of high-risk tumors. Renal masses located in the upper pole of the kidney, especially those that are complex in nature and high risk, pose a challenge for surgical management. Especially considering the TRIFECTA Criteria; in such cases, partial nephrectomy performed with an eleventh rib resection can be considered a reliable surgical option with a shorter ischemia time, supporting the preservation of long-term renal function.

## Data Availability

In our country, it is forensically necessary to obtain the consent of the patients regarding the availability of the data. Unfortunately, the availability of all data cannot be ensured due to the lack of patient consent.
